# Normal adrenal and thyroid function in patients who survive COVID-19 infection

**DOI:** 10.1210/clinem/dgab349

**Published:** 2021-05-19

**Authors:** Sophie A Clarke, Maria Phylactou, Bijal Patel, Edouard G Mills, Beatrice Muzi, Chioma Izzi-Engbeaya, Sirazum Choudhury, Bernard Khoo, Karim Meeran, Alexander N Comninos, Ali Abbara, Tricia Tan, Waljit S Dhillo

**Affiliations:** 1 Division of Diabetes, Endocrinology and Metabolism, Department of Metabolism, Digestion and Reproduction, Imperial College London, London, UK; 2 Department of Endocrinology, Imperial College Healthcare NHS Trust, London, UK; 3 Department of Clinical Biochemistry, Imperial College Healthcare NHS Trust, London, UK; 4 Department of Endocrinology, Division of Medicine, Faculty of Medical Sciences, Royal Free Campus, University College London, London, UK

**Keywords:** COVID-19, SARS-CoV-2, adrenal insufficiency, adrenal function, thyroid function, thyroid gland

## Abstract

**Context:**

The COVID-19 pandemic continues to exert an immense burden on global health services. Moreover, up to 63% of patients experience persistent symptoms, including fatigue, after acute illness. Endocrine systems are vulnerable to the effects of COVID-19 as many glands express the ACE2 receptor, used by the SARS-CoV-2 virion for cellular access. However, the effects of COVID-19 on adrenal and thyroid gland function after acute COVID-19 remain unknown.

**Objectives:**

Our objectives were to evaluate adrenal and thyroid gland function in COVID-19 survivors.

**Design:**

A prospective, observational study was undertaken.

**Setting:**

Clinical Research Facility, Imperial College NHS Healthcare Trust.

**Participants:**

Seventy patients ≥ 18 years at least 3 months after diagnosis of COVID-19 were included.

**Intervention:**

Participants attended a research study visit (08:00-09:30), during which a short Synacthen test (250 µg IV bolus), and thyroid function assessments were performed.

**Results:**

All patients had a peak cortisol ≥450 nmol/l after Synacthen, consistent with adequate adrenal reserve. Basal and peak serum cortisol did not differ according to disease severity or history of dexamethasone treatment during COVID-19. There was no difference in baseline or peak cortisol after Synacthen or in thyroid function tests, or thyroid status, in patients with fatigue (n=44) compared to those without (n=26).

**Conclusions:**

Adrenal and thyroid function ≥3 months after presentation with COVID-19 was preserved. Whilst a significant proportion of patients experienced persistent fatigue, their symptoms were not accounted for by alterations in adrenal or thyroid function. These findings have important implications for the clinical care of patients after COVID-19.

## Introduction

As the COVID-19 pandemic continues [1], questions regarding its consequences on the endocrine system remain [2]. The route by which SARS-CoV-2, the virus responsible for COVID-19, accesses cells (via ACE2 receptors) renders the endocrine system potentially susceptible to damage by COVID-19, as ACE2 receptors are expressed in the adrenal gland, thyroid gland and testes [3,4]. Some evidence of the adverse effects on endocrine function by coronavirus infections comes from SARS, the precursor to COVID-19, caused by another coronavirus, SARS-CoV. Post-mortem examination of patients with SARS revealed SARS-CoV RNA in the pituitary gland [5], in addition to destruction of thyroid follicular and parafollicular cells [6], and a longitudinal study of survivors of SARS showed that up to 39% had hypocortisolism [7]. In patients with COVID-19, microscopic adrenal lesions in post mortem specimens [8,9], adrenal haemorrhage [10,11] and infarction [12] have been reported. There is also the potential for iatrogenic impairment of adrenal function as therapeutic glucocorticoid treatment has been routinely used since July 2020 to treat patients with COVID-19 requiring oxygen therapy or ventilatory support [13], raising the possibility of tertiary adrenal insufficiency [14].

It is increasingly evident that the health impact of COVID-19 extends beyond the initial infection, with up to 63% of patients reporting ongoing symptoms including fatigue [15,16]. The pathophysiology of this 'post-COVID syndrome' is currently poorly understood. Adrenal and thyroid gland dysfunction is known to be associated with fatigue [17], and may additionally present with other components of the post-COVID syndrome, including autonomic dysfunction and cognitive impairment [18,19]. As these endocrinopathies are eminently treatable, ~~and hence~~ it is imperative to identify any contribution they may have to the persistent symptoms experienced by patients after COVID-19 infection.

Therefore, our objectives in this study were to evaluate adrenal and thyroid function in survivors of COVID-19, and to investigate the relationship between them and the symptom of persistent fatigue experienced post-COVID. We addressed this by investigating adrenal and thyroid function prospectively in patients ≥3 months after initial diagnosis of COVID-19.

## Methods

### Ethical approval

This study was performed in accordance with the Declaration of Helsinki. All participants provided written informed consent prior to inclusion in the study. Ethical approval for this study was granted by the London Bridge Research Ethics Committee (REC ref 20/HRA/4110). This study was registered with ISRCTN (ISRCTN15615697).

### Study design and Participants

In this prospective study, participants were recruited from a cohort of patients who attended Imperial College London NHS Healthcare Trust with a clinical suspicion of COVID-19 between March-November 2020. Additional participants were also recruited via adverts placed in social media for patients who had tested positive for COVID-19 ≥3 months prior to their inclusion in the study. All patients aged ≥18yrs with a diagnosis of COVID-19 confirmed using either real-time RT-PCR testing of a nasopharyngeal swab, confirmatory imaging (chest radiograph or CT scan), or a positive serum SARS-CoV-2 IgG antibody test taken after symptom onset were eligible for inclusion. Patients who were prescribed steroids (oral, inhaled, topical or intra-articular) following recovery from COVID-19, and those taking other medications known to affect cortisol-binding globulin (including oral oestrogens) were excluded from the study.  Similarly, those patients with underlying health conditions or states known to influence cortisol-binding globulin (including pregnancy, end-stage renal failure, or underlying malignancy) were also excluded.

**Study protocol**

Participants were invited to attend for their study visit at least 3 months following their initial presentation with COVID-19. Participants were non-fasted and study visits commenced between 08:00-09:30am. Study visits comprised a clinical assessment, including medical history and physical examination, and a urinary pregnancy test in women of reproductive age. During the clinical assessment, patients were asked regarding the presence of persistent symptoms following COVID-19 (including cough, shortness of breath, chest pain, low-grade fever, fatigue, headache, difficulty concentrating, muscle aches, loss of appetite, nausea, diarrhoea, rash, or change in mood). In the absence of a validated measure of fatigue in patients post-COVID-19 at the time of study design, rates of fatigue were initially quantified using the 'yes/no' response to the direct question of experience of fatigue, consistent with other studies investigating COVID-19 [20,21] and other disease paradigms [22,23]. All participants were subsequently asked to rate the frequency of fatigue experienced following acute COVID-19 on a scale of 0-4 (0=none of the time, 1=a little of the time, 2=about half of the time, 3=most of the time, 4=all of the time) and severity of fatigue on a scale of 0-4 (0=symptom not present, 1=mild, 2=moderate, 3=severe, 4=very severe). The severity of COVID-19 was classified by applying WHO classification criteria [24] to parameters from participants' presentation with COVID-19 by two independent clinicians (SAC and MP) who were not involved in the clinical care of participants during their presentation with COVID-19. Where discrepancies occurred, the least severe classification was selected. Additionally, severity was also assessed according to the level of care provided. A cannula was inserted and baseline samples taken for serum cortisol, thyroid stimulating hormone (TSH), free thyroxine (fT4) and free triiodothyronine (fT3), and plasma adrenocorticotropic hormone (ACTH). Additionally, samples were also taken for SARS-CoV-2 antibody testing. Following this, 250µg Tetracosactide (Synacthen) was injected intravenously. Samples for cortisol were taken at 30 and 60 minutes after injection.

**Interpretation of Short Synacthen test**

Participants were considered to have an adequate response to Synacthen if they reached a peak cortisol value of ≥450 nmol/l and increment by ≥150 nmol/l from baseline, either at 30 or 60 minutes after Synacthen [18,25]. Although a lower reference limit of peak cortisol ≥430nmol following 250µg Synacthen has been proposed for the Abbott cortisol assay [25], our local policy is to use a more conservative cortisol cut-off of 450nmol/l to denote adequate response.

**Assay methodology**

Serum cortisol, TSH, fT4 and fT3 were measured using Abbott Alinity ci-series analyser using chemiluminescent microparticle immunoassays. The lower limits of detection were as follows: cortisol 19.3 nmol/l, TSH 0.01 mU/l, fT4 5.4 pmol/l, fT3 1.46 pmol/l. Interassay coefficients of variation were: cortisol ≤5.1%, TSH ≤2.1%, fT4 ≤3%, fT3 ≤4.8%. Intraassay coefficients of variation were: cortisol ≤4.3%, TSH ≤2.1%, fT4 ≤3%, fT3 ≤3.8%. Serum ACTH was measured using Siemens Immulite. The inter- and intra-assay coefficient of variation was ≤10%. Reference ranges were as follows: TSH 0.3-4.2 mU/l, fT4 9-23 pmol/l, fT3 2.5-5.7 pmol/l. Serum antibodies to SARS-CoV-2 N protein (IgG) were measured using Abbott Architect assay. For those with indeterminate result, additional testing for antibodies to the receptor binding domain of SARS-CoV-2 spike protein (IgG) was performed using Imperial Hybrid DABA [26].

**Outcomes**

The primary outcome of this observational study was the number of patients with an insufficient response to intravenous Synacthen 250µg IV ≥3 months after presentation with COVID-19 [25]. The secondary outcomes were the TSH, fT4 and fT3 levels measured ≥3 months after presentation with COVID-19. Post-hoc subgroup comparisons were made for differences in those reporting persistent fatigue on direct questioning (vs those who did not) and those who received dexamethasone as part of their treatment for COVID-19 (vs those who did not).

**Statistical analysis**

Data were analysed using GraphPad Prism version 9.0. Data distribution was assessed using D'Agostino and Pearson, Kolmogorov-Smirnov tests and Q-Q plots. Parametric data were presented as mean ± standard deviation (SD), whereas non-parametric data were presented as median with interquartile range (IQR). Continuous data that were parametrically distributed were compared using a Student's t-test for two groups, or one-way analysis of variance (ANOVA) for three or more groups. Continuous data that were non-parametrically distributed were compared using Mann-Whitney U tests for two groups, or Kruskal-Wallis test with post hoc Dunn's test for three or more groups. Categorical data were compared using chi-squared test. Relationship between two continuous variables was determined using Pearson's correlation, for parametrically distributed data, and Spearman's rank correlation for non-parametrically distributed data.

**Results**

*Baseline Characteristics*

In total, 110 survivors of COVID-19 presenting in March-November 2020 were considered for inclusion to this study, of whom 40 were excluded either due to taking medications or conditions known to interfere with the short Synacthen test (n=6) or because they declined to take part (n=34) (Figure 1), resulting in 70 participants being enrolled in the study. The mean age (±SD) was 55.9 (±13.0) yrs, and 67.1% of the study population were male (Table 1). Of this cohort, 77.1% of patients had been hospitalized; 22.0% of those hospitalized had also required either non-invasive ventilation or intensive care treatment (Table 1), and 31.4% received dexamethasone as part of their acute treatment for COVID-19. The median (IQR) duration of admission was 5 (1, 8) days. Patients attended for their research study visit appointment at a median (IQR) of 210 (112, 261) days following presentation (Table 1). Those who received dexamethasone attended their clinical research appointment sooner than those who did not (median (IQR) days following presentation: no dexamethasone 242 (209.5, 287.0), dexamethasone 95.5 (88.8, 113.3) $P < .001$) reflecting the fact that dexamethasone treatment was mandated in U.K. protocols for treatment of COVID-19 from July 2020 onwards. On direct questioning, 62.9% (n=44) of patients reported fatigue.

*Adrenal function is preserved after COVID-19*

At follow-up ≥3 months after initial presentation with COVID-19, mean (±SD) baseline cortisol was 233.1 (±77.5) nmol/l with a median (IQR) basal ACTH of 14.85 (8.34, 22.10) ng/l. All patients achieved a peak cortisol of ≥450 nmol/l post Synacthen (Figure 2), with a mean cortisol at 30-minutes post Synacthen of 562.1 (87.6) nmol/l and mean cortisol at 60-minutes post-Synacthen of 636.6 (97.6) nmol/l (Figure 2A). Baseline and peak cortisol (nmol/l) were not related to time from admission (baseline cortisol vs time from admission Pearson ρ -0.09, *P*=.45; peak cortisol vs time from admission Pearson ρ -0.09, P=0.46; Supplemental Figures 1A and B [27] https://doi.org/10.14469/hpc/8118). Of those with available data (n=24), there was no relationship between cortisol taken within the first 48 hrs of acute admission with COVID-19, and either baseline cortisol or peak cortisol following Synacthen at follow-up endocrine testing (baseline cortisol vs admission cortisol Pearson ρ 0.21, *P*=.34; peak cortisol vs admission cortisol Pearson ρ 0.24, *P*=.25; Supplemental Figures 1C and D [27] https://doi.org/10.14469/hpc/8118). Subgroup analysis did not reveal any difference in response to Synacthen with disease severity, as determined by WHO classification (Table 2, Figure 1C), level of care received (Figure 1D) or antibody status (Figure 1E).

Treatment protocols mandating dexamethasone for the treatment of COVID-19 requiring supplemental oxygen or assisted ventilation were introduced in the UK in June 2020, following initial study design. A post-hoc analysis of cortisol response in patients who received dexamethasone (n=22), did not reveal any difference in cortisol response at either 30- or 60-minutes post-Synacthen compared to those who did not (Table 2, Figure 3A and B). Similarly, basal cortisol was not different between the two groups (Figure 3C).

*Adrenal function is preserved in patients with persistent fatigue following COVID-19*

In our cohort, post hoc analysis revealed that 62.9% (n=44) of patients reported persistent fatigue on direct questioning ≥3 months after presentation with COVID-19 (Fatigue cohort), with median (IQR) time since presentation of 205 (102, 281) days (Table 1). In the Fatigue cohort there was a slight predominance of male patients reflecting the composition of the cohort as a whole (male gender in total cohort 67.1% versus 54.5% in the Fatigue cohort), but in the No fatigue cohort, there was a more marked predominance of male patients (No fatigue 88.5% vs Fatigue 54.5%, $P$=.004). The patients in the Fatigue cohort were younger (mean ± SD age 52.8 ±12.9 yrs, vs 61.1 ±11.6 yrs for the No fatigue cohort, $P$=.009) (Table 1). In the fatigue cohort, 54.5% had had severe COVID-19 illness, compared to 23.1% of those in the No fatigue cohort, and 50.0% had received dexamethasone during their treatment, compared to none of those without fatigue (Table 1). There was no difference in serum cortisol or change in serum cortisol from baseline at any time-point following Synacthen between the Fatigue and No Fatigue cohorts (Figure 4A, Table 2). Peak cortisol at either 30 or 60 minutes following Synacthen also did not differ between the two groups (Figure 4B). There was no correlation between either baseline cortisol or peak cortisol and fatigue severity or frequency (Figure 4C-E). Additionally, median (IQR) ACTH concentrations were not different between those who did not receive dexamethasone, compared to those who did (no dexamethasone 16.05 (9.05, 22.13) ng/L; dexamethasone 14.05 (7.53, 21.75), $P$ = .54). No significant differences between the ACTH concentrations were seen between the Fatigue and No fatigue cohorts:  12.35 (7.55, 21.55) ng/L and 19.15 (14.03, 22.80) respectively, $P$=.06.

*Thyroid function after COVID-19*

In patients without pre-existing thyroid disease (n=68), TSH, fT4 and fT3 were within the reference range for all patients attending ≥3 months after presentation with COVID-19 (median (IQR) TSH 1.32 (0.97, 2.09) mU/l, fT4 12.30 (11.65, 13.08) pmol/l, fT3 4.40 (4.08, 4.80) pmol/l (see Table 2). In the Fatigue cohort, serum TSH, fT4 and fT3 did not differ from the No fatigue cohort (Table 2, Figure 5A and B).

**Discussion**

To our knowledge, this is the first detailed assessment of adrenal and thyroid function in patients ≥3 months following COVID-19 infection. Our group recently published data demonstrating that in the acute setting, a single cortisol measurement taken within 48 hrs of admission predicted mortality. We showed that there was an appropriate stress response during acute COVID-19 without any suggestion of acute adrenal insufficiency [28]. However, given that adrenal insufficiency can occur following critical illness [29] we wished to determine whether patients who have survived COVID-19 have any evidence of adrenal gland dysfunction. In our study cohort, all patients had a normal response to Synacthen, achieving a peak cortisol ≥450 nmol/l, regardless of the severity of COVID-19, their antibody status, or whether they had received dexamethasone. Basal and peak cortisol levels did not differ by either disease severity, nor by whether they experienced persistent fatigue following COVID-19. Following publication of the data from the RECOVERY trial, the use of dexamethasone became standard care for all patients admitted with COVID-19 who had an oxygen requirement. Data from our cohort suggests that administration of dexamethasone according to this protocol (i.e. 6 mg once daily for a maximum of 10 days) [30] does not impair adrenal function in the medium-term, a reassuring observation given the large number of patients who will have received this regimen as part of routine treatment of COVID-19. Our data is also reassuring given that another coronavirus (SARS) has been reported to be associated with a prevalence of hypocortisolism up to 39% in a sample of a similar size (n=61) [7]. It should be noted that these authors used an older radioimmunoassay for cortisol compared to the Abbott immunoassay in this study which has been validated against gold-standard GC-MS methods [25]. Moreover, the definition of hypocortisolism used in the earlier study included either an 8 am cortisol of 138 nmol/l or a peak cortisol of 550 nmol/l after 1µg Synacthen. We elected to use the 250µg Synacthen test to assess hypothalamic-pituitary-adrenal axis integrity. This is a frequently utilised test, with widely accepted, assay-specific reference ranges recommended for detection of primary adrenal insufficiency [18]. Whilst it has been suggested that the 1µg Synacthen test may be more sensitive at detecting secondary adrenal insufficiency [31], several meta-analyses have failed to demonstrate a difference between the two tests [32–34]. Furthermore, significant variability exists in the methodology used to draw up the 1 µg Synacthen dose, raising the possibility of inaccuracies in undertaking the test and its consequent diagnostic performance[35]. The standard 250µg Synacthen test remains the most widely used dynamic function test for assessing the integrity of the HPA axis [35,36], and is the gold-standard test for diagnosing primary adrenal insufficiency [18,35,37]. Given the pathophysiology of COVID-19, primary, secondary and tertiary adrenal insufficiency were all distinct clinical possibilities, and thus the standard SST provided the most practical way to interrogate the HPA axis in its entirety. Importantly, both the 250µg and 1µg Synacthen tests represent supraphysiological stimuli to the adrenal glands, with both often resulting in higher cortisol responses than the physiological stressor of hypoglycaemia [31], and with Mayenknecht and colleagues demonstrating 1 µg Synacthen to result in maximal stimulation of the adrenal glands [38]. Thus, the 250 µg Synacthen test afforded us the most appropriate assessment of the HPA axis in its entirety, replicating standard clinical practice.

Persistent symptoms following COVID-19 are reported to affect approximately 53-63% of patients [15,16] and indeed this is reflected in our cohort, where direct questioning revealed that 62.9% of patients experienced persistent fatigue following COVID-19.

Not only is disturbance of the adrenal axis frequently associated with fatigue, but dysfunction of the thyroid axis may frequently present with tiredness. Of those patients not already known to have thyroid disease (n=68), thyroid function tests (TSH, fT4 and fT3) were all within the normal range. We have also previously shown that there is a small reduction, on average, of TSH and FT4 compared to the pre-COVID-19 baseline and that there appears to be a recovery to intra-individual set-points for fT4 and TSH [39]. Moreover, there was no evidence of increase in frequency of hypo- or hyperthyroidism in those patients with fatigue, compared to those without.

This study does have some limitations. During the development of this study, details of persistent symptoms including fatigue following COVID-19 were emerging [40]. In the absence of any validated measure for fatigue post-COVID-19, we adopted a pragmatic approach, quantifying rates of fatigue using the 'yes/no' response to the direct question of experience of fatigue, consistent with both studies investigating COVID-19 [20,21] and other disease paradigms [22,23]. We then used an ordinal scale to quantify both the frequency of fatigue and severity of fatigue, however such an approach, as with other measures of fatigue, is subjective. In addition, due to the single assessment of patients, we are unable to provide longitudinal data for all patients included in the study. For patients with details of their initial admission with COVID-19 illness available, we have provided data on cortisol levels taken within the first 48hrs of admission. Furthermore, given that all patients demonstrated adequate adrenal reserve and thyroid levels at over 3 months after initial illness, it is unlikely that clinically significant endocrine abnormalities would develop on more prolonged follow up. Additionally, by excluding patients on glucocorticoid treatment, as well as other medications known to affect cortisol-binding globulin (like the combined oral contraceptive pill), it may be that some patients with adrenal and thyroid dysfunction following COVID-19 were not included in the study. However, given that administration of steroids is known to influence thyroid function tests, and interpretation of the response to Synacthen, it was necessary to exclude such patients to prevent the misclassification of patients who have survived COVID-19.

In summary, in this prospective study we assessed for the presence of adrenal and thyroid dysfunction in patients at least 3 months following COVID-19. We have shown that all patients in our cohort had adequate adrenal reserve irrespective of disease severity and dexamethasone treatment. In keeping with the literature, we have shown that a significant number of patients experience persistent fatigue after COVID-19. However, we report for the first time that these symptoms are not accounted for by overt adrenal or thyroid gland dysfunction. These findings have important implications for the clinical assessment of patients after COVID-19, an emerging field of medicine.

**Acknowledgements**

***Financial Support:*** This work was supported by the National Institute for Health Research (NIHR) and the NIHR Imperial Clinical Research Facility and NIHR Imperial Biomedical Research Centre at Imperial College Healthcare NHS Trust. The views expressed are those of the authors and not necessarily those of the NHS, the NIHR or the Department of Health and Social Care. The Section of Endocrinology and Investigative Medicine is funded by grants from the UK Medical Research Council (MRC), Biotechnology and Biological Sciences Research Council, and NIHR. S.C. and M.P. are funded by NIHR Clinical Lectureships. T.T. is funded by the NIHR and the JP Moulton Charitable Foundation; B.K. is funded by the JP Moulton Charitable Foundation; E.G.M. is funded by an MRC Clinical Research Training Fellowship (MR/T006242/1); B.P. is funded by an Imperial Health Charity Fellowship; C.I. is funded by an Imperial College-BRC Post-doctoral Post-CCT Fellowship; A.N.C. is funded by the NHS; A.A. is funded by an NIHR Clinician Scientist Award (CS-2018-18-ST2-002); and W.S.D. is funded by an NIHR Professorship (RP-2014-05-001).

***Author Contributions:*** Conceptualizing of the study: S.A.C., B.K., K.M., S.C. A.N.C., A.A., T.T, and W.S.D.; collecting and recording of the demographic, clinical, and laboratory data: S.A.C., M.P, E.G.M., B.P., B.M., C.I.; analyzing the data: S.C. M.P, A.A; drafting of the manuscript: S.C., W.S.D.; and revising of all subsequent versions of the manuscript: S.C. and W.S.D. All authors read and approved the final manuscript.

***Data Availability:*** Some or all data sets generated during and/or analyzed during the present study are not publicly available but are available from the corresponding author on reasonable request.

**Table Legends**

**Table 1: Baseline characteristics of patients attending ≥ 3 months following presentation with COVID-19.** Data are means (SD) for continuous variables parametrically distributed and medians (lower quartile, upper quartile) for continuous non-parametrically distributed variables. For categorical data, numbers of patients (percentages) are presented. Continuous data that are parametrically distributed were compared using t-tests, continuous data not parametrically distributed were compared using Mann-Whitney U test. Groups of continuous data parametrically distributed were compared using ANOVA test. Groups of continuous data non-parametrically distributed were compared using Kruskal-Wallis test. Categorical data were compared using Chi-Squared test. NIV: non-invasive ventilation, ITU: intensive therapy unit, Conv. Plasma: convalescent plasma, Other: additional treatments include Anakinra (interleukin-1 receptor antagonist), Kaletra (Lopinavir/Ritonavir), Namilumab, NA; not applicable.

**Table 2: Hormonal data of patients attending ≥ 3 months following presentation with COVID-19.** Data are means (SD) for continuous variables parametrically distributed and medians (lower quartile, upper quartile) for continuous variables non-parametrically distributed. For categorical data, numbers of patients (percentages) are presented. Continuous data that are parametrically distributed were compared using t-tests, continuous data not parametrically distributed were compared using Mann-Whitney U test. Groups of data parametrically distributed were compared using ANOVA test. Groups of data non-parametrically distributed were compared using Kruskal-Wallis test. TSH: Thyroid stimulating hormone, fT4: free thyroxine, fT3: free triiodothyronine, no Dex: no dexamethasone treatment, Dex: dexamethasone.

**Figure Legends**

**Figure 1: STROBE diagram showing patients meeting inclusion criteria (n=70).**

**Figure 2: Serum cortisol response to Synacthen 250µg in patients 3 months after COVID-19 (n=70).** **(A)** Individual responses of serum cortisol at 30 and 60 minutes after an IV bolus of 250 µg of Synacthen in patients ≥3 months after presentation with COVID-19 are presented. (B) Individual responses in change in serum cortisol from baseline at 30 and 60 minutes after an IV bolus of 250 µg of Synacthen in patients assessed ≥3 months after presentation with COVID-19 are presented. **(C)** Mean and SD change in cortisol from baseline (nmol/l) after an IV bolus of 250µg of Synacthen in patients recovering from COVID-19 by WHO Disease Severity (mild, n=12 represented by green symbols and line; moderate, n=30, represented by orange symbols and lines; severe, n=21, represented by red symbols and line; critical, n=7, represented by purple symbols and line). **(D)** Mean (error bars show SD) change in cortisol from baseline (nmol/l) after Synacthen 250µg in patients recovering from COVID-19 by level of care required: community care (n=16), represented by blue symbols and line; hospitalised and not requiring ventilatory support (n=45), represented by red symbols and lines; ventilatory support required (n=9), represented by black symbols and lines. **(E)** Mean and SD change in cortisol from baseline (nmol/l) after Synacthen 250 µg in patients recovering from COVID-19 by antibody status as determined by Abbott Architect IgG to SARS-CoV-2 (detected, n=44, represented by blue symbols and lines; not detected/indeterminate, n=16, represented by red symbols and lines).

**Figure 3: Serum cortisol response to 250µg Synacthen in patients who received dexamethasone as part of their treatment for COVID-19 (n=22, represented by red symbols and lines) vs those who did not (n=48, represented by black symbols and lines). (A)** Data presented are mean (error bars show SD) cortisol (nmol/l) at time 30-minutes and 60-minutes following Synacthen 250 µg. **(B)** Data presented are mean (error bars show SD) change in serum cortisol from baseline (nmol/l) at time 30-minutes and 60-minutes following Synacthen 250 µg. **(C)** Graph of baseline serum cortisol (nmol/l).

.

**Figure 4: Serum cortisol response to 250µg Synacthen in patients 3 months after COVID-19 with persistent fatigue (n=44) compared to those without (n=26).**

**Figure 4A:** Mean (error bars show SD) for serum cortisol (nmol/l) at time 30-minutes and 60-minutes following Synacthen comparing patients with persistent fatigue (n=44, red lines and symbols) compared to those with no persistent fatigue (n=26, blue lines and symbols).

**Figure 4B:** Graph of peak serum cortisol in patients with persistent fatigue and those without persistent fatigue. Individual data points plotted with horizontal line representing mean, whiskers represent SD.

**Figure 4C:** Graph of baseline cortisol in groups classified by tiredness frequency graded 0 (not present), 1 (present a little of the time), 2 (present about half of the time), 3 (present most of the time), 4 (present all of the time).

**Figure 4D:** Graph of peak cortisol in groups classified by tiredness frequency graded 0 (not present), 1 (present a little of the time), 2 (present about half of the time), 3 (present most of the time), 4 (present all of the time).

**Figure 4E:** Graph of baseline cortisol in groups classified by tiredness severity graded 0 (not present), 1 (mild), 2 (moderate), 3 (severe), 4 (very severe). (F) Graph of peak cortisol (nmol/l) in groups classified by tiredness severity score graded 0 (not present), 1 (mild), 2 (moderate), 3 (severe), 4 (very severe).

**Figure 5: Thyroid function tests in patients with persistent fatigue.**

**Figure 5A:** Graph of individual TSH (mU/l) values in patients with fatigue (n=44) compared to those without (n=24), in those with no pre-existing thyroid disease (n=68). Individual data points plotted with horizontal line representing median, whiskers represent upper and lower interquartile range. The normal reference range for TSH using Abbott Architect assay (0.3-4.2mU/l) is depicted using the red dashed line. LLRR, lower limit reference range; ULRR, upper limit reference range.

**Figure 5B:** Graph of individual fT4 (pmol/l) values in patients with fatigue (n=44) compared to those without (n=24), in those with no pre-existing thyroid disease (n=68). Individual data points plotted with horizontal line representing median, whiskers represent upper and lower interquartile range. The normal reference range for fT4 using Abbott Architect assay (9-23pmol/l) is depicted using the red dashed line. LLRR, lower limit reference range; ULRR, upper limit reference range.

**Table 1: Baseline characteristics of patients attending ≥ 3 months following presentation.**

| Participant characteristics | Total Cohort (n=70) | No Dexamethasone (n=48) | Dexamethasone (n=22) | *P* value | No fatigue (n=26) | Fatigue (n=44) | *P* value |
|---|---|---|---|---|---|---|---|
| **Age (years)** | 55.9 (13.0) | 55.6 (13.3) | 56.6 (12.6) | .77 | 61.1 (11.6) | 52.8 (12.9) | .009 |
| ***Sex:*** | | | | | | | |
| **Male** | 47/70 (67.1%) | 33/48 (68.8%) | 14/22 (63.6%) | .79 | 23/26 (88.5%) | 24/44 (54.5%) | .004 |
| **Female** | 23/70 (32.9%) | 15/48 (31.3%) | 8/22 (36.4%) | | 3/26 (11.5%) | 20/44 (45.5%) | |
| ***Ethnicity:*** | | | | | | | |
| **Asian** | 17/70 (24.3%) | 7/48 (14.6%) | 10/22 (45.5%) | .006 | 8/26 (30.8%) | 9/44 (20.5%) | .82 |
| **Black** | 7/70 (10.0%) | 4/48 (8.3%) | 3/22 (13.6%) | | 3/26 (11.5%) | 4/44 (9.1%) | |
| **Mixed** | 2/70 (2.9%) | 1/48 (2.1%) | 1/22 (4.6%) | | 1/26 (3.9%) | 1/44 (2.3%) | |
| **Other – not stated** | 17/70 (24.3%) | 17/48 (35.4%) | 0 (0.0%) | | 5/26 (19.2%) | 12/44 (27.3%) | |
| **White** | 27/70 (38.6%) | 19/48 (39.6%) | 8/22 (36.4%) | | 9/26 (34.6%) | 18/44 (40.9%) | |
| ***Comorbidities:*** | | | | | | | |
| **Hypertension** | 24/70 (34.3%) | 13/48 (27.1%) | 11/22 (~~0.~~50.0%) | .05 | 6/26 (23.1%) | 18/44 (40.9%) | .20 |
| **Cardiovascular disease** | 4/70 (34.3%) | 4/48 (8.3%) | 0/22 (0.0%) | | 3/26 (11.5%) | 1/44 (2.3%) | |
| **Diabetes** | 36/70 (14.3%) | 26/48 (54.2%) | 10/22 (45.5%) | | 14/26 (53.9%) | 22/44 (50.0%) | |
| **Type 1** | 1/36 (2.7%) | 0/26 (0.0%) | 1/10 (10.0%) | | 0/14 (0.0%) | 1/22 (4.5%) | |
| **Type 2** | 18/36 (22.2%) | 10/26 (38.5%) | 8/10 (80.0%) | | 6/14 (42.9%) | 12/22 (54.5%) | |
| | 17/36 | 16/26 | 1/10 | | 8/14 | 9/22 | |

| | | | | | | | |
|---|---|---|---|---|---|---|---|
| **Unspecified** | (47.2%) | (61.5%) | (10.0%) | | (57.1%) | (40.9%) | |
| **Obesity (BMI >30kg/m$^2$)** | | | | | | | |
| **Yes** | 15/70 (21.4%) | 6/48 (12.5%) | 9/22 (40.9%) | | 4/26 (15.4%) | 11/44 (25.0%) | |
| **No** | 30/70 (42.9%) | 20/48 (41.7%) | 10/22 (45.5%) | | 17/26 (65.4%) | 13/44 (29.5%) | |
| **Unknown** | 25/70 (35.7%) | 22/48 (45.8%) | 3/22 (13.6%) | | 5/26 (19.2%) | 20/44 (45.5%) | |
| ***Smoking status:*** | | | | | | | |
| **Current smoker** | 2/70 (2.9%) | 1/48 (2.1%) | 1/22 (4.5%) | .23 | 2/26 (7.7%) | 0/44 (0.0%) | .16 |
| **Ex-smoker** | 6/70 (8.6%) | 6/48 (12.5%) | 0/22 (0.0%) | | 3/26 (11.5%) | 3/44 (6.8%) | |
| **Never-smoked** | 60/70 (85.7%) | 39/48 (81.3%) | 21/22 (95.5%) | | 21/26 (80.8%) | 39/44 (88.6%) | |
| **Unknown** | 2/70 (2.9%) | 2/48 (4.2%) | 0/22 (0.0%) | | 0/0 (0.0%) | 2/44 (4.6%) | |
| ***Disease outcome:*** | | | | | | | |
| **Hospitalized** | 54/70 (77.1%) | 32/48 (66.7%) | 22/22 (100.0%) | NA | 17/26 (65.4%) | 37/44 (84.1%) | .07 |
| **NIV** | 5/54 (9.3%) | 1/32 (3.1%) | 4/22 (18.2%) | | 0/26 (0.0%) | 5/37 (13.5%) | |
| **ITU admission** | 7/54 (13.00%) | 3/32 (9.4%) | 4/22 (18.2%) | | 3/26 (11.5%) | 4/37 (10.8%) | |
| **Non-hospitalized** | 16/70 (22.9%) | 16/48 (33.3%) | 0/22 (0.0%) | | 9/26 (34.6%) | 7/44 (15.9%) | |
| ***Disease Severity:*** | | | | | | | |
| **Mild** | 12/70 (17.1%) | 12/48 (25.0%) | 0/22 (0.0%) | .04 | 8/26 (30.8%) | 4/44 (9.1%) | .03 |
| **Moderate** | 30/70 (42.9%) | 19/48 (39.6%) | 11/22 (50.0%) | | 6/26 (23.1%) | 24/44 (54.5%) | |

| | | | | | | | |
|---|---|---|---|---|---|---|---|
| **Severe** | 21/70 (30.0%) | 14/48 (29.2%) | 7/22 (31.8%) | | 9/26 (34.6%) | 12/44 (27.3%) | |
| **Critical** | 7/70 (10.0%) | 3/48 (6.3%) | 4/22 (18.2%) | | 3/26 (11.5%) | 4/54 (7.4%) | |
| **Dexamethasone treatment** | 22/70 (31.4%) | NA | 22/22 (100%) | NA | 0/26 (0.0%) | 22/44 (50.0%) | NA |
| **Cumulative dose dexamethasone treatment (mg)** | 38.73 (18.49) | NA | 38.73 (18.49) | NA | NA | 38.73 (18.49) | NA |
| ***Additional treatments:*** | | | | | | | |
| **Remdesivir** | 15/70 | 1/48 (2.1%) | 14/22 (63.6%) | .05 | 8/26 (30.8%) | 7/44 (15.9%) | .26 |
| **Tocilizumab** | 1/70 | 1/48 (2.1%) | 0/22 (0.0%) | | 1/26 (3.9%) | 0/44 (0.0%) | |
| **Conv. Plasma** | 2/70 | 0/48 (0.0%) | 2/22 (9.1%) | | 1/26 (3.9%) | 1/44 (2.3%) | |
| **Other** | 3/70 | 1/48 (2.1%) | 2/22 (9.1%) | | 0/26 (0.0%) | 3/44 (6.8%) | |
| **Duration of admission (days)** | 5.0 (1.0, 8.0) | 4.0 (0.0, 8.0) | 6.0 (4.0, 9.8) | <.001 | 6.5 (3.0, 1.3) | 4.0 (0.0, 7.8) | .03 |
| **Time since presentation (days)** | 210.0 (112.0, 261.0) | 242.0 (209.5, 287.0) | 95.5 (88.8, 113.3) | <.001 | 215.00 (121.0, 252.3 | 209.0 (102.0, 281.0) | .83 |

**Table 1: Baseline characteristics of patients attending ≥3 months following presentation with COVID-19.** Data are means (SD) for continuous variables parametrically distributed and medians (lower quartile, upper quartile) for continuous non-parametrically distributed variables. For categorical data, numbers of patients (percentages) are presented. Continuous data that are parametrically distributed were compared using t-tests, continuous data not parametrically distributed were compared using Mann-Whitney U test. Groups of continuous data parametrically distributed were compared using ANOVA test. Groups of continuous data non-parametrically distributed were compared using Kruskal-Wallis test. Categorical data were compared using Chi-Squared test. NIV: non-invasive ventilation; ITU: intensive therapy unit, Conv Plasma: convalescent plasma; Other: additional treatments include Anakinra (interleukin-1 receptor antagonist), Kaletra (Lopinavir/Ritonavir), Namilumab; NA: not applicable.

**Table 2: Hormonal Data of patients attending ≥3 months following presentation with COVID-19.**

| | Total Cohort (n=70) | No Dex (n=48) | Dex (n=22) | *P* value | No Fatigue (n=26) | Fatigue (n=44) | *P* value | Mild (n=12) | Moderate (n=30) | Severe (n=21) | Critical (n=7) | *P* value |
|---|---|---|---|---|---|---|---|---|---|---|---|---|
| **Baseline Cortisol (nmol/l)** | 233.1 (77.5) | 229.3 (75.7) | 241.6 (82.5) | 0.54 | 243.8 (84.9) | 227.2 (73.5) | .40 | 216.9 (64.2) | 224.8 (85.0) | 268.2 (72.4) | 191.3 (44.7) | .06 |
| **30-min Cortisol (nmol/l)** | 562.1 (87.6) | 549.3 (93.1) | 591.2 (66.6) | 0.81 | 551.4 (63.5) | 568.1 (98.9) | .45 | 549.1 (135.2) | 571.5 (84.8) | 562.5 (59.1) | 543.9 (84.5) | .83 |
| **60-min Cortisol (nmol/l)** | 636.6 (97.6) | 623.3 (105.5) | 665.8 (71.4) | 0.78 | 623.0 (89.9) | 644.2 (101.8) | .34 | 629.3 (127.3) | 648.0 (101.0) | 632.5 (79.1) | 613.0 (90.5) | .83 |
| **30-min Change in** | 328.8 (102.4) | 320.0 (103.9) | 348.7 (98.6) | 0.29 | 307.7 (87.7) | 340.7 (109.1) | .20 | 332.2 (130.0) | 346.5 (96.9) | 294.3 (87.9) | 352.6 (109.8) | .31 |

| | | | | | | | | | | | | |
|---|---|---|---|---|---|---|---|---|---|---|---|---|
| **Cortisol (nmol/l)** | | | | | | | | | | | | |
| **60-min Change in Cortisol (nmol/l)** | 403.5 (112.30 | 394.0 (119.3) | 424.2 (94.6) | 0.30 | 379.2 (112.1) | 417.0 (111.4) | .18 | 412.4 (110.4) | 423.1 (116.0) | 364.3 (103.2) | 421.7 (119.8) | .30 |
| **ACTH (ng/l)** | 14.85 (8.34, 22.10) | 16.05 (9.05, 22.13) | 14.05 (7.53, 21.75) | 0.54 | 19.15 (14.03, 22.80) | 12.35 (7.55, 21.55) | .06 | 9.35 (6.38, 12.43) | 17.75 (7.48, 22.25) | 21.20 (11.75, 27.10) | 15.00 (8.30, 20.40) | .01 |
| **TSH (mU/l)** | 1.32 (0.97, 2.09) | 1.42 (1.02, 2.13) | 1.24 (0.80, 1.82) | >0.99 | 1.33 (1.03, 2.09) | 1.36 (0.96, 2.10) | .95 | 1.60 (0.90, 2.53) | 1.29 (1.06, 1.79) | 1.32 (0.84, 2.07) | 1.24 (1.03, 3.03) | .84 |
| **fT4 (pmol/l)** | 12.30 (11.65, 13.08) | 12.20 (11.75, 12.73) | 12.40 (11.55, 13.60) | 1.00 | 12.30 (11.68, 13.30) | 12.25 (11.65, 13.03) | .65 | 12.05 (11.60, 12.70) | 12.30 (11.80, 12.90) | 12.40 (11.83, 13.60) | 11.20 (10.60, 12.30) | .15 |
| **fT3 (pmol/l)** | 4.40 (4.08, 4.80) | 4.40 (4.00, 4.80) | 4.30 (4.18, 4.63) | 1.00 | 4.40 (4.20, 4.80) | 4.20 (3.90, 4.70) | .14 | 4.15 (3.93, 4.48) | 4.30 (3.90, 4.83) | 4.40 (4.15, 4.80) | 4.60 (4.20, 4.70) | .34 |
| | **Admission Cohort** | **No Dex (n=24)** | **Dex (n=0)** | ***P*** | **No Fatigue (n=11)** | **Fatigue (n=13)** | ***P*** | **Mild (n=0)** | **Moderate (n=14)** | **Severe (n=9)** | **Critical (n=1)** | ***P*** |

| | (n=24) | | | value | | | value | | | | | value |
|---|---|---|---|---|---|---|---|---|---|---|---|---|
| **Admission cortisol (nmol/l)** | 566 (428.5, 749.3) | 566 (428.5, 749.3) | NA | NA | 650.0 (505.0, 1194.0) | 489.0 (407.5, 657.5) | .04 | NA | 472.5 (393.8, 555.5) | 760.0 (664.0, 1205) | 1158 | NA |

**Table 2: Hormonal data of patients attending ≥3 months following presentation with COVID-19.** Data are means (SD) for continuous variables parametrically distributed and medians (lower quartile, upper quartile) for continuous variables non-parametrically distributed. For categorical data, numbers of patients (percentages) are presented. Continuous data that are parametrically distributed were compared using t-tests, continuous data not parametrically distributed were compared using Mann-Whitney U test. Groups of data parametrically distributed were compared using ANOVA test. Groups of data non-parametrically distributed were compared using Kruskal-Wallis test. ACTH; Adrenocorticotropic hormone, TSH; Thyroid stimulating hormone, fT4; free thyroxine, fT3; free triiodothyronine, no Dex; no dexamethasone treatment, Dex; dexamethasone. NA; not applicable.

**Figure 1**

uscrip
Accept

**Figure 2**

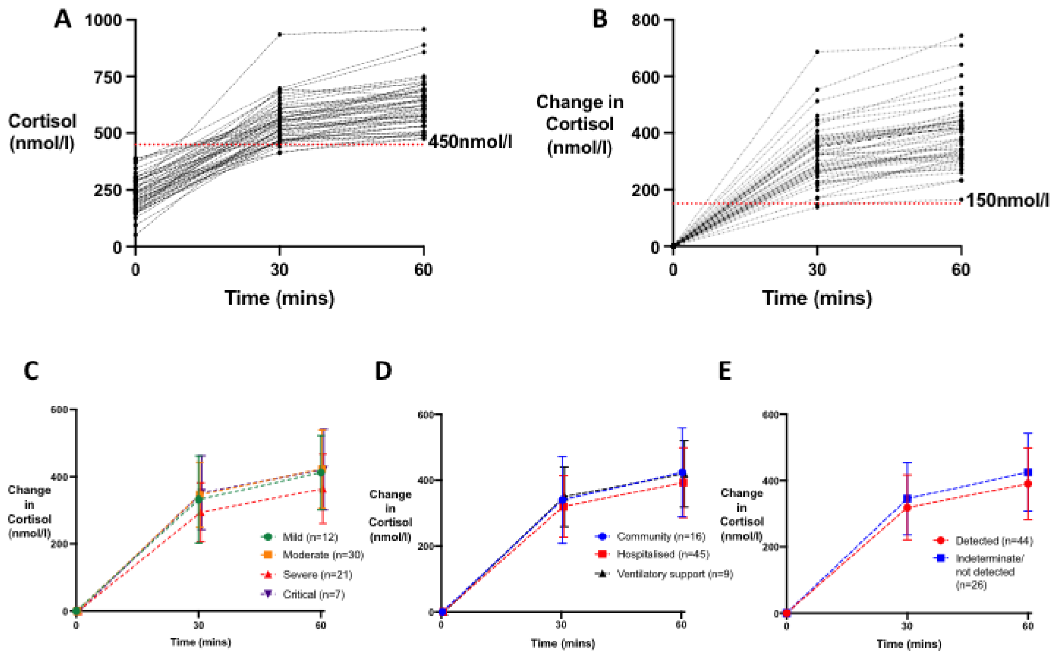

**Figure 3**

A
800
600
400
200
0
Cortisol (nmol/l)
Dexamethasone (n=22)
No dexamethasone (n=48)
0
30
60
Time (mins)
B
600
400
200
0
Change in Cortisol (nmol/l)
Dexamethasone (n=22)
No dexamethasone (n=48)
0
30
60
Time (mins)
C
500
400
300
200
100
0
Baseline Cortisol (nmol/l)
No dex (n=48)
Dex (n=22)

Figure 4

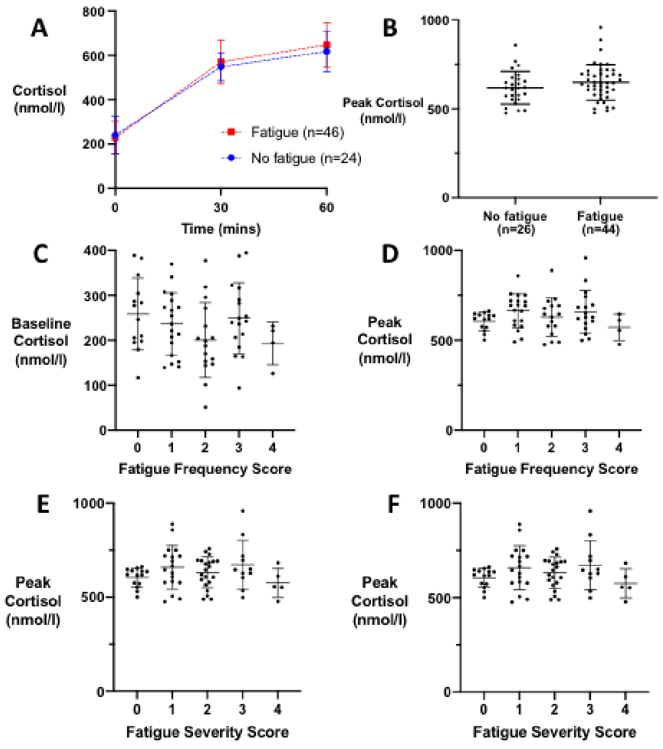

Accepte scrip

Figure 5

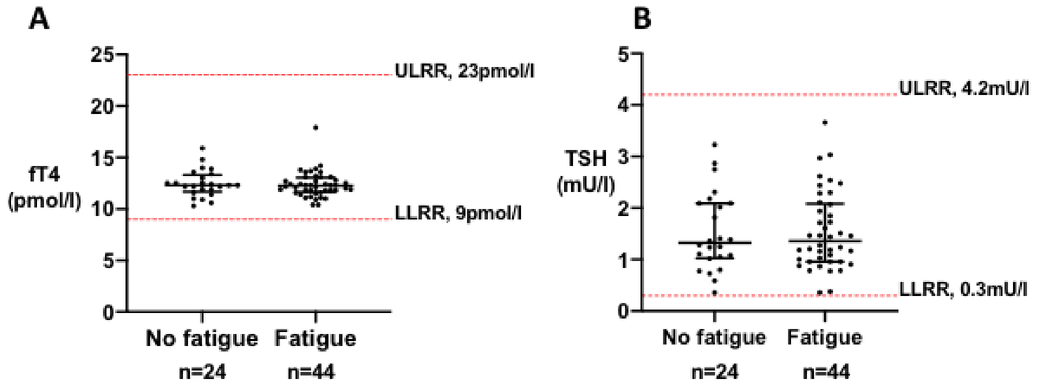

A
25
20
15
10
5
0
fT4
(pmol/l)
ULRR, 23pmol/l
LLRR, 9pmol/l
No fatigue
n=24
Fatigue
n=44
B
5
4
3
2
1
0
TSH
(mU/l)
ULRR, 4.2mU/l
LLRR, 0.3mU/l
No fatigue
n=24
Fatigue
n=44
